# Editorial: Advanced Catalysts in Ring-Opening Polymerization of Cyclic Esters

**DOI:** 10.3389/fchem.2019.00404

**Published:** 2019-06-04

**Authors:** Jincai Wu, Hsuan-Ying Chen, Pimpa Hormnirun

**Affiliations:** ^1^State Key Laboratory of Applied Organic Chemistry, Key Laboratory of Nonferrous Metal Chemistry and Resources Utilization of Gansu Province, College of Chemistry and Chemical Engineering, Lanzhou University, Lanzhou, China; ^2^Department of Medicinal and Applied Chemistry, Kaohsiung Medical University, Kaohsiung, Taiwan; ^3^Department of Chemistry, National Sun Yat-Sen University, Kaohsiung, Taiwan; ^4^Department of Medical Research, Kaohsiung Medical University Hospital, Kaohsiung, Taiwan; ^5^Department of Chemistry, Kasetsart University, Bangkok, Thailand

**Keywords:** ring-opening polymerization, cyclic esters, caprolactone (CL), lactide, O-carboxyanhydrides, butyrolactone, decalactone

Now lots of biodegradable polymers were produced to replace the petroleum-based plastics to solve the environmental pollution. Herein, the scientists designed and synthesized the effective catalysts for the ring-opening polymerization (ROP) of cyclic esters, such as *o*-carboxyanhydrides, ε-caprolactone (CL), β-butyrolactone (BBL), ε-decalactone (DL), and lactide (LA). The catalysts for the ROP of *o*-carboxyanhydrides were explored in the review by Zhong and Tong. The ligands are essential for enhancing the catalytic activity and the selectivity control of catalysts. A study of CL polymerization by using Al complexes as catalysts reported by Zou et al. revealed that Al complexes bearing biphenolate phosphinoxide had higher catalytic activity of CL and *rac*-LA ROP than those bearing amino-bridged and phosphine-bridged biphenolates. The Zn complexes bearing amido-pyridinates reported by Chen et al. revealed that Zn complex with chelating dimethyl amino group could be tolerated in tetrahydrofuran during CL polymerization and exhibited greater catalytic activity than that with chelating pyridinyl group. The *rac*-LA polymerization mediated by urea/potassium alkoxide system reported by Kan et al. showed that urea/potassium alkoxide system had very high catalytic activity (90% conversion, 1–2 min), and produced PLAs with high P_i_ values (P_*i*_ = 0.83–0.93) and narrow M_w_/M_n_ values (1.05–1.09). Studies of BBL and DL copolymerizations employing (salan) yttrium catalysts reported by Kiriratnikom et al. revealed that the syndiotactic-enriched poly(3-hydroxybutyrate)s blocks copolymers were produced. Finally, a special case reported by Lu et al. revealed that the phenoxyl oxygen of the catalyst attracked the α-hydrogen of the LA to produce the weaken H-O bond, and the benzyl alcohol initiated LA directly without replacing the ethyl group of the Zn complex.

The Research Topic of Advanced Catalysts in Ring-Opening Polymerization of Cyclic Esters provides knowledge on how the ligands affect the catalytic activity of the catalysts and a new possible mechanism of ROP. It is useful information for the scientists to design more effective catalysts for ROP of cyclic esters.

## Author Contributions

All authors listed have made a substantial, direct and intellectual contribution to the work, and approved it for publication.

### Conflict of Interest Statement

The authors declare that the research was conducted in the absence of any commercial or financial relationships that could be construed as a potential conflict of interest.

